# Indications, sub-types and complications of surgically treated thyroid disease in Africa: A systematic review and meta-analysis

**DOI:** 10.1016/j.sopen.2025.06.006

**Published:** 2025-06-18

**Authors:** Bekalu Getachew, Mekbeb Afework, Girmaye Tamrat

**Affiliations:** a Department of Biomedical Science, Jimma university, Jimma, Ethiopia; b Department of Human Anatomy, School of Medicine, College of Health Sciences, Addis Ababa university, Addis Ababa, Ethiopia; c Department of Surgery, School of Medicine, College of Health Sciences, Addis Ababa University, Addis Ababa, Ethiopia

**Keywords:** Patterns, Thyroidectomy, Surgical management, Complications, Africa, Thyroid disease

## Abstract

**Objectives:**

Thyroidectomy is a surgical procedure that reduces or removes the thyroid gland the aim of this systematic review and meta-analysis was to assess the pooled prevalence and sub-types of thyroidectomy and characterize its postoperative complications in some low and middle income African countries.

**Methods:**

The studies were identified through an exhaustive search of reputable databases Twenty-two studies were selected based on the inclusion and exclusion criteria. Data were extracted using a standardized and pre-tested data extraction checklist, and the analysis was done using STATA version 14 statistical software. Heterogeneity was assessed using I^2^ statistics.

**Result:**

Toxic goiters were the most common indication for thyroidectomy accounting for 46.62 % of cases. Cosmetic reasons (41.07 %) and suspicion of malignancy (11.30 %) were the other common indications. Regarding surgical procedures, sub-total thyroidectomy (39.27 %) was the predominant surgical procedure, followed by lobectomy and isthmusectomy (34.88 %) and near-total thyroidectomy (34.77 %) respectively. The pooled prevalence of postoperative complications following thyroidectomy was 26.6 % [95%CI, 18.3–34.89]. Hypoparathyroidism (8.49 %) was the most common complication, followed by recurrent laryngeal nerve injury (7.96 %) and dysphonia (7.28 %).

**Conclusion:**

A toxic goiter was the most common indication for thyroidectomy. The pooled prevalence of postoperative complications was comparably higher than international figures. Hypoparathyroidism was the predominant postoperative complication.

## Introduction

The thyroid gland comprises right and left lobes situated anterolateral to the larynx and trachea. [1,2]. The spectrum of thyroid diseases includes simple goiter, toxic goiter, thyroiditis, adenoma and cancers [3,4]. The delicate anatomy of the anterior neck and its surrounding critical structures make thyroidectomy a challenging procedure to perform safely and effectively [5]. Thyroidectomy involves restoring the functional changes resulting from enlargement and pressure on the trachea, hyperfunction or malignant invasion, although cosmesis is the most common indication [6]. Although thyroidectomy is generally a safe procedure, it carries some risk of complications such as bleeding, tension hematoma, postoperative infection, damage to the recurrent laryngeal nerve, and damage to the parathyroid gland [7].

In France, about 50,000 patients undergo thyroidectomy yearly [[8], [9], [10]]. In Germany, about 100,000 thyroid operations were performed; in the year 2012, there were 44,000 total thyroidectomies and 42,000 subtotal resections performed [11], and the most common indication was bilateral multinodular goiter [[12], [13], [14]]. A study conducted in Sudan revealed that more than half 52 % of thyroidectomies were partial, followed by hemithyroidectomy (26 %) and sub-total thyroidectomy (11 %) [15,16]. In Kenya, the majority of operations on the thyroid were total thyroidectomy (35.9 %), followed by near-total(30.7 %) and sub-total thyroidectomies (10 %) [17].

A study conducted in Ethiopia showed the types of thyroidectomies as lobectomy and isthmusectomy (24 %), near total thyroidectomy (14 %) and lobectomy alone (7.8 %). Only 4.2 % of the patients had a total thyroidectomy, and most (50 %) of thyroid operations were sub-total [18]. Still, the current trend in most countries, especially in developed countries, is more to near-total and total thyroidectomies [19]. The two most common early complications of thyroid surgery are hypocalcemia (20 %–30 %) and recurrent laryngeal nerve (RLN) injury (5 %–11 %) [20]. The complications usually arise following sub-total and total thyroidectomy [[21], [22], [23]]. Even though there is much research done on surgical indications and complications of thyroidectomy in diverse African countries, pooled data is lacking in low and middle income African countries, so this systematic review and meta-analysis were done to assess the pooled prevalence and sub-types of thyroidectomy and characterize its postoperative complications in some low and middle income African countries.

## Methods

### Description of search strategies

The article search was conducted in Scopus, EMBASE, PubMed, and Cochrane Library. The leading search terms were following: “Low and middle income African countries ” “thyroid disease”, “surgical management”, “goiter”, “sub-types” “thyroidectomy”, “indications”, “post-thyroidectomy”, “Complications”.

### Eligibility criteria

**Fig. 1:**
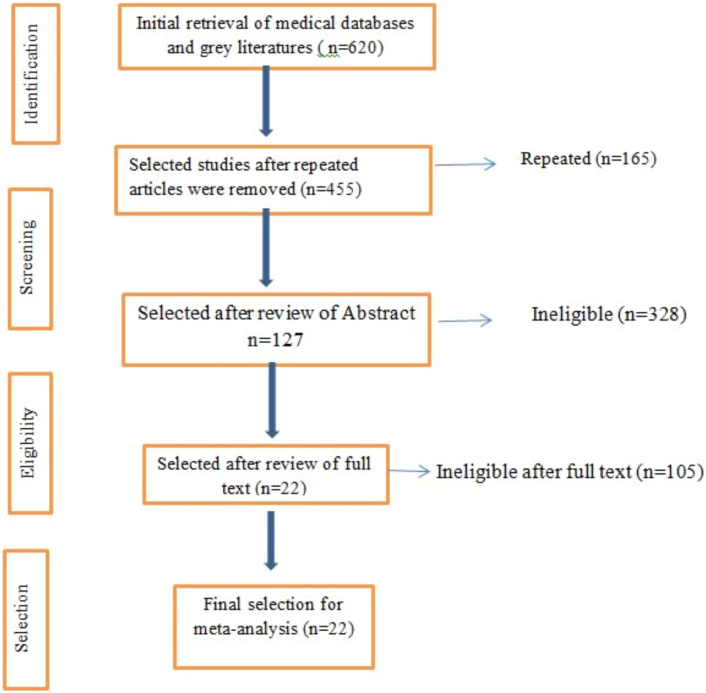
Flowchart on selection of systematic review and meta-analysis of thyroid disease in Some African Countries, 2022.

Original studies and those reported in English were included. Articles published from 1995 to 2022 G.C and studies conducted on low income and middle income African countries among adults aged 18 years were included. (Fig. 1).

### Summary of studies selected for review

The title and abstracts were reviewed; one hundred twenty seven articles were selected after abstract review. Full abstract was available for twenty two studies (Table 1).

**Table 1:** Characteristics of surgically treated thyroid disease in Some African Countries,2022.

S.n	Authors	Sample size	Country	Year	Study type	Quality using JBI
1	Nyeki, A.R.N et al.	56	Gabon	2012	Prospective	Medium
2	Wondossen et al.	199	Ethiopia	2022	Prospective	Medium
3	Ersumo, T. et al.	213	Ethiopia	2020	Retrospective	High
4	L.N Wagana	61	Kenya	2002	Retrospective	High
5	Ouattara, M.A… et al.	53	Mali	2015	Retrospective	Medium
6	Mohamed Mustafa	49	Sudan	2013	Prospective	Medium
7	UOsimeand MN Okobia	50	Nigeria	2004	Retrospective	High
8	Ali, N.and Naa'Ya, H·U	184	Nigeria	2009	Retrospective	High
9	Chalya, P.L et al.	144	Tanzania	2011	Prospective	High
10	Tonga, N.L., et al.	48	Nigeria	2022	Retrospective	High
11	Kpolugbo, J et al.	80	Nigeria	2012	retrospective	High
12	Abebe Bekele and Mensur Osman	75	Ethiopia	2006	Prospective	High
13	Abebe, B. et al.	137	Ethiopia	2001	Retrospective	High
14	Dodiyi-Manuel, A., & Dodiyi-Manuel, S. T.	80	Nigeria	2016	Retrospective	High
15	Yisak S and Engida A.	222	Ethiopia	2020	Retrospective	Medium
16	Adejumo, A et al.	79	Nigeria	2022	retrospective	High
17	Samuel, S.A. and Rebecca, S·H	72	Nigeria	2019	Retrospective	High
18	Regonne, P.E.J et al.	90	Senegal	2016	Retrospective	High
19	Burali G et al.	137	Uganda	2016	Prospective	High
20	Dakubo, J.C·B et al.	528	Ghana	2013	Prospective	High
21	Niyirera, E. et al.	44	Rwanda	2021	Retrospective	High
22	J.Ramos… et al.	211	Ethiopia	2013	Retrospective	High

### Research questions(hypothesis) (PIO)

Population: Adult patients aged 18 years and above.

Intervention: Thyroidectomy.

Outcome: subtypes and complications following surgery.

### Data extraction

The three authors (B.G, M.A. and G.T) meticulously and independently extracted all necessary data using a comprehensive data extraction template on Microsoft Excel.

### Heterogeneity and publication bias

Publication bias and heterogeneity were checked using Funnel plot and Egger's tests. A *p*-value of less than 0.05 was considered to declare the statistical significance. The heterogeneity of studies was also assessed using I^2^ test statistics. A random effect model was used for those with heterogeneity.

### Data analysis/synthesis of results

The data were extracted using Microsoft Excel 2016 format and analyzed using STATA version 14.0. A sub-group analysis was conducted to identify the possible source of heterogeneity. Sensitivity analysis and Meta-regression was also done.

### Quality appraisal

The quality of each article was meticulously assessed by two independent reviewers using the Joanna Briggs Institute (JBI) critical appraisal tool. Only studies scoring 50 % were considered for systematic review and meta-analysis.

## Results

After an extensive search, 22 studies were identified. Two thousand eight hundred twelve (2812) patients' operations were performed on the thyroid gland.

### Indications for thyroidectomy

#### Meta-analysis on common indications for thyroidectomy

The pooled prevalence of most typical indications of thyroidectomy in our study sample was toxic goiters (46.62 %) with significant heterogeneity (I^2^ = 99.9 %), followed by cosmetic reasons (41.07 %) (I^2^ = 99.8 %) and suspicion of malignancy (11.3 %) (I^2^ = 66.3 %), (Table 2).

**Table 2:** A systematic review and meta-analysis on indications for thyroidectomy in some Africa countries, 2022.

Indications	Effect	95 % CI		Heterogeneity(I^2)^	Number of studies
Compression symptoms	10.44 %	7.51 %	13.37 %	0.0 %	4
Suspension of malignancy	11.30 %	4.00 %	18.60 %	66.3 %	2
Cosmetic reasons	41.07 %	3.19	78.95	99.8 %	3
Toxic goiter	46.62 %	−3.63	96.87	99.9 %	3
Recurrence	6.54 %	0.29	12.80	0.0 %	2

#### Publication bias for indication of toxic goiter

**Fig. 2:**
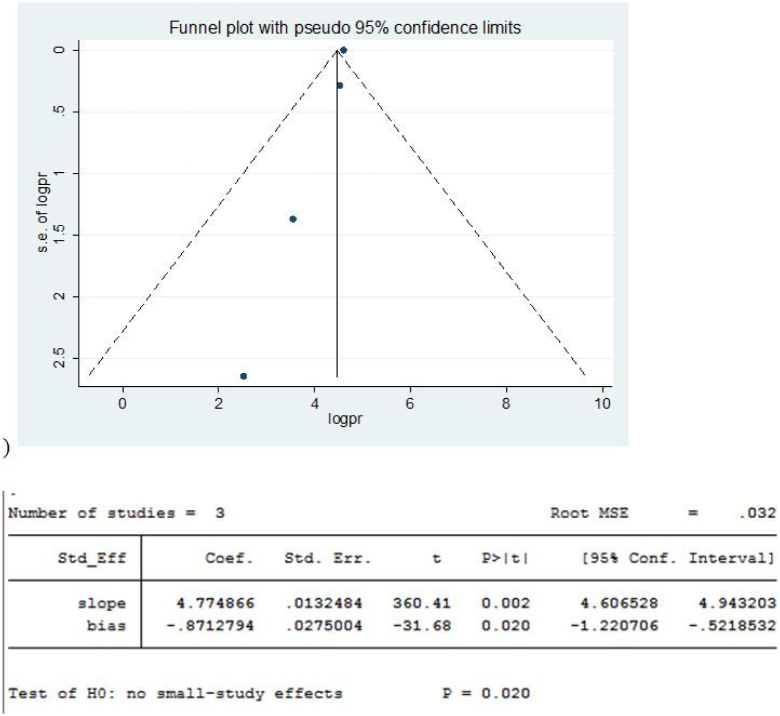
Results of Funnel plot and Egger Test for Toxic goiter in some African countries, 2022.

Based on the results, the funnel plot looks asymmetric, and the egger's test result was significant (*P* = 0.020), indicating a significant publication bias (Fig. 2).

### Sub-types of thyroidectomy

#### Meta-analysis on pooled prevalence of sub-total thyroidectomy

**Fig. 3:**
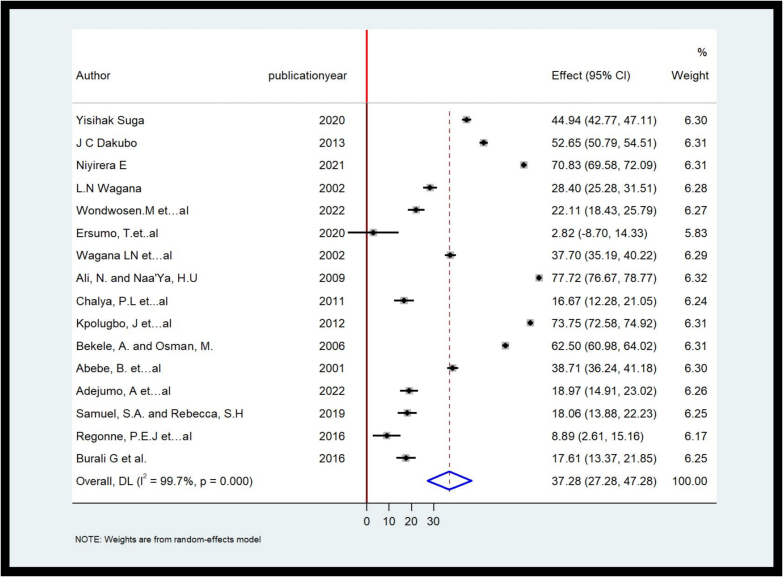
Forest plot on the pooled prevalence (frequency) of sub-total thyroidectomy in some African countries, 2022.

The pooled prevalence of sub-total thyroidectomy in our study sample was 37.28 % [95%CI, 27.28 %–47.28 %]. The forest plot also indicated variations in the prevalence of sub-total thyroidectomy in some African countries (Fig. 3).

#### Meta-analysis on the prevalence(frequency) of lobectomy and isthmusectomy

**Fig. 4:**
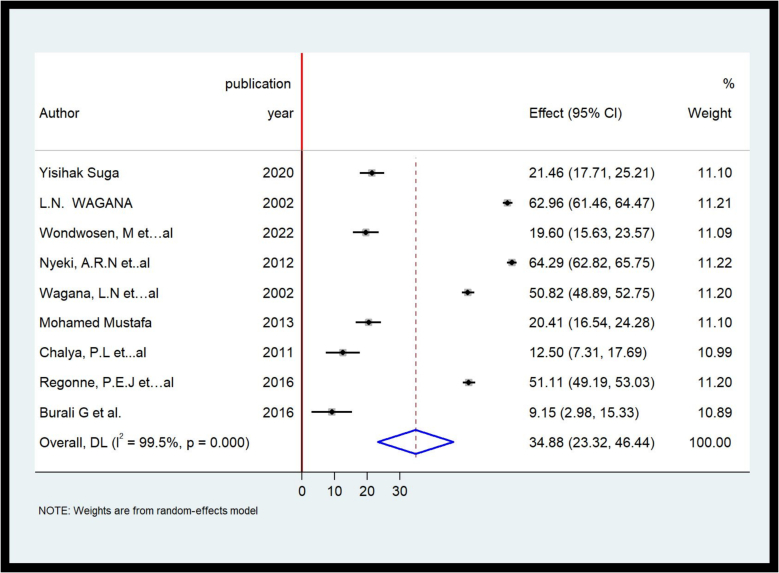
Forest plot on pooled frequency of Lobectomy and Isthmusectomy in some African countries, 2022.

The overall pooled prevalence of lobectomy and isthmusectomy was 34.88 % [95%CI, 23.32 %-46.44 %].(Fig. 4).

##### Sub-group analysis

According to the sub-group Analysis, The highest pooled estimate or frequency of lobectomy and isthmusectomy was reported from Western Africa (55.42 %) with significant heterogeneity (I^2^ = 98.8 %) of studies.

Meta-regression. A random effect meta regression was run by region, the analysis indicated that there was significant heterogeneity by region (East Africa = 0.037).

#### Meta-analysis on prevalence of lobectomy

**Fig. 5:**
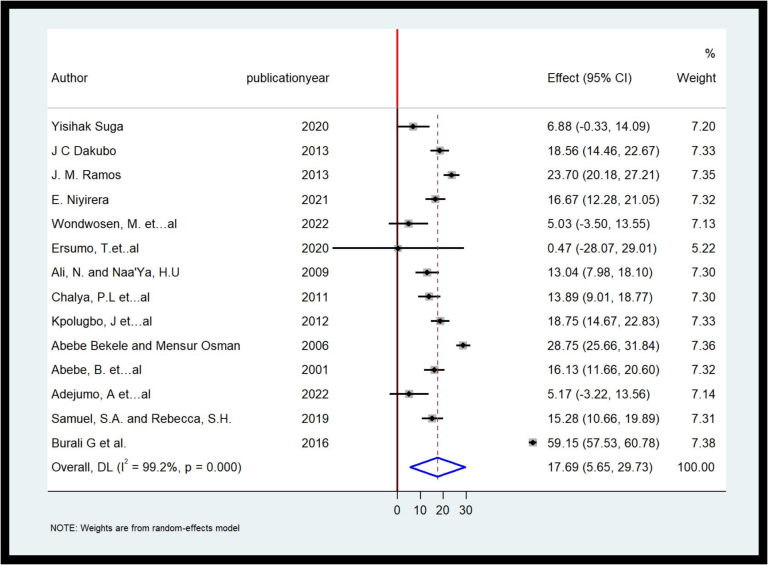
Forest plot on the pooled frequency of Lobectomy in Some African Countries, 2022.

The pooled frequency of hemi or partial lobectomy was 17.69 % [95%CI, 5.65 %–29.73 %]. (Fig. 5).

#### Meta-analysis on prevalence of total thyroidectomy

**Fig. 6:**
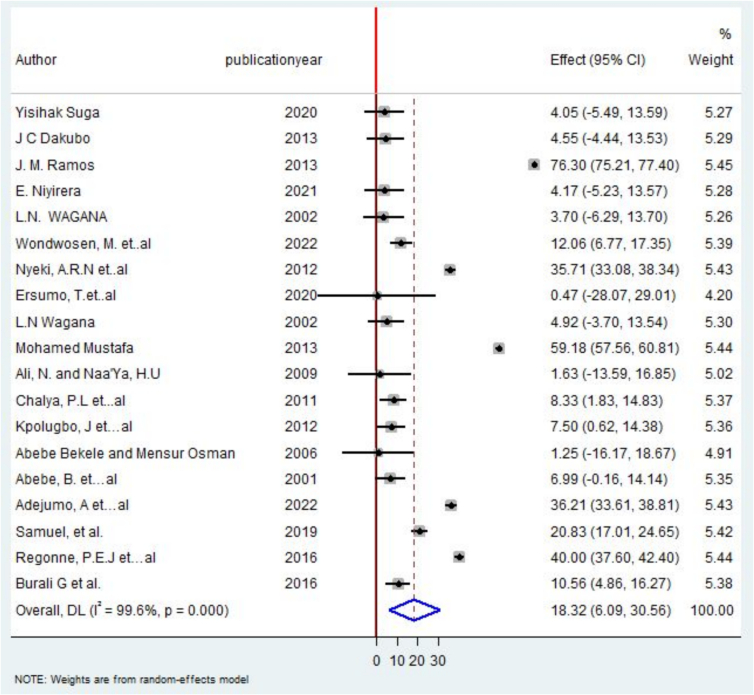
Forest plot on the pooled frequency of total thyroidectomy in Some African Countries, 2022.

The pooled prevalence of total thyroidectomy in our study sample was 18.32 % [95 %CI, 6.08 %–30.55 %). Significant heterogeneity was found (I^2^ = 99.6 %) (Fig. 6).

#### Near-total thyroidectomy

**Fig. 7:**
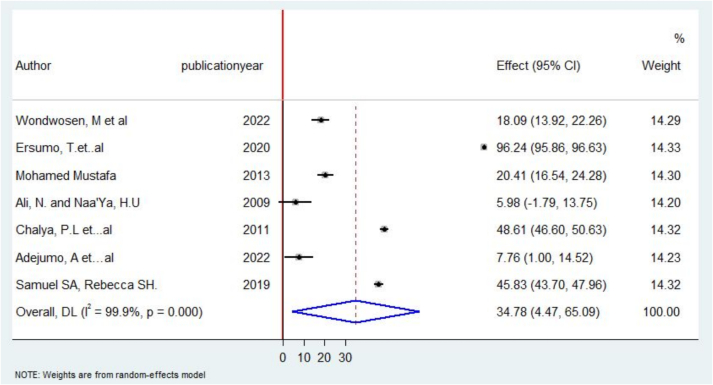
Forest plot on near-total thyroidectomy in Some African Countries, 2022.

The pooled frequency of near-total thyroidectomy was 34.77 % [95 % CI, 4.46–65.08 %] (Fig. 7).

### Postoperative complications following thyroidectomy

**Fig. 8:**
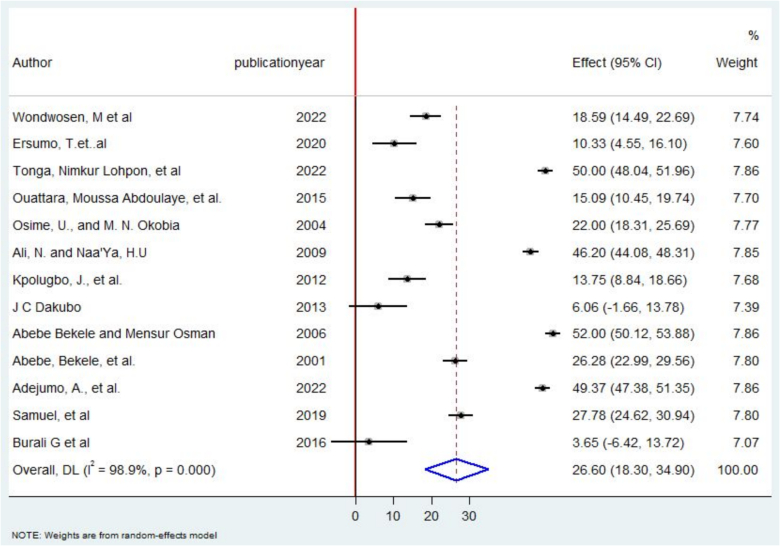
A forest plot on post-operative complications following thyroidectomy in Some African Countries, 2022.

The overall pooled prevalence of post-operative complication following thyroidectomy was reported to be 26.60 % [95%CI, 18.30–34.90 %] in Low and middle income African countries.(Fig. 8).

#### Common post-operative complications

The most common complication following post-operative thyroidectomy procedure were hypoparathyroidism (8.49 %) followed by recurrent laryngeal nerve injury (7.96 %) and dysphonia(7.28 %) (Table 3).

**Table 3:** Pooled prevalence of common post-operative complications following thyroidectomy in Some African Countries, 2022.

Common complications	Effect	95 % CI		Heterogeneity(I^2)^)	Number of studies
Recurrence	1.32	−10.66 %	13.30 %	0 %	2
Recurrent laryngeal nerve injury	7.96	−3.89	19.82	94.5 %	9
Hemorrhage	6.83	−2.35	16.03	55 %	5
Hematoma	4.22	0.48	7.96	0 %	8
Dysphonia	7.28	4.00	10.55	0 %	7
Hypothyroidism	4.10	−0.57	8.78	0 %	6
Hypoparathyroidism	8.49	−0.09	17.06	69 %	4
Wound site infection	5.10	1.25	8.95	0 %	6
Hypocalcemia	5.6	1.64	9.56	0 %	5

## Discussion

According to this systematic review and meta-analyses, the most common indication for thyroidectomy in our study sample was toxic goiter (46.62 %), followed by cosmetic reasons (41.07 %) and suspicion of malignancy (11.30 %). Consistent findings were reported from a study conducted elsewhere [24]. The higher pooled prevalence of toxic goiter in the study was explained previously by authors, who indicated that iodine deficiency is the leading cause and is most prevalent in most parts of the continent. A higher prevalence of toxic goiters might be related to improvement in the living standard and universal iodization programme of salt that could potentially lead to toxic adenoma or a toxic multinodular goiter [25]. According to the results on funnel plot and eagers test, there is a significant publication bias on all of the reported indications of thyroidectomy indicating that the individual studies have possibly reported only significant results. The most common type of thyroidectomy in our study sample was sub-total thyroidectomy (37.28 %), followed by lobectomy isthmusectomy (34.88 %) and near-total thyroidectomy (34.77 %). The magnitude of sub-total thyroidectomy was higher than previously conducted research in Bangladesh (10 %) [26]. The higher prevalence of sub-total thyroidectomy in the region might be due to the variations in the pathologic cause of the thyroid disease and differences in postoperative follow-up and treatment. Surgeries other than sub-total thyroidectomy require continuous patient status monitoring as well as meticulous postoperative lifelong thyroid hormone replacement therapy. This adds a significant burden as there is a lack of follow-up and medication availability, and Africa is struggling in the health sector. Hence, sub-total thyroidectomy might be preferred. The other possible explanation is that general surgeons do most thyroidectomies in this Africa regions to reduce post-thyroidectomy complications, and the approach may be more conservative. The current finding is lower than a previously reported research from Turkey (58.3 %) [27]. The possible reason might be the difference in surgery indications, sample size, socioeconomic status, and population structure. However, lobectomy and isthmusectomy were considered to be the most often practiced surgical intervention for thyroid in some African countries. These procedures completely spare the contralateral lobe. This has a greater role in ensuring thyroid function and leaving the contralateral surgical field intact, thereby reducing surgical risks in the case of a recurrence. Near-total thyroidectomy was shown to be the third most common procedure practiced in our meta-analysis. Total thyroidectomy needs to be conducted in a way that preserves the parathyroid glands, recurrent laryngeal nerves and bleeding. Total thyroidectomy is the least to be done in developing countries [28]. According to the current systematic review and meta-analysis, only 18.32 % of thyroidectomies were total. Based on the report from the forest plot, there is a significant heterogeneity on meta-analysis of all of the sub-types of the thyroidectomy, the possible reason for the variation might be due to the difference in statistical reliability between the studies.

The pooled prevalence of postoperative complications following thyroidectomy was found to be 26.6 % in low and middle income African countries. This finding is comparable with findings from Spain (21 %) [29] and lower than previously reported research from Bangladesh (17 %) [26], and The finding was higher than previously reported data from an American university college (3.28 %) [30]. The higher prevalence of complications could be related to the variations in the surgical staff between the countries and differences in the reporting of complications that could potentially overestimate the pooled prevalence. Hypoparathyroidism (8.49 %) was the leading cause of complication following thyroidectomy in the current study. The pooled prevalence was lower than previous study conducted in Nigeria (16.675 %) [31]. The aetiology could be that surgical manipulations in the parathyroid gland could potentially cause transient hypoparathyroidism. The current meta-analysis also found that the pooled prevalence of recurrent laryngeal nerve injury following thyroidectomy was 7.96 %. our result was higher than a previous study reported the incidence between 0 % and 3.6 % after thyroidectomy [32]. Evidence suggests that recurrent laryngeal nerve (RLN) injuries represent one of the most feared complications after thyroid and parathyroid surgery [33]. The nerve injury mechanisms include complete or partial transection, traction or handling of the nerve, contusion, crush, burn, clamping, misplaced ligature, and compromised blood supply [34]. A meta-analysis that included 14,934 patients showed an incidence of 3.4 % regarding recurrent laryngeal nerve paralysis; the incidence was higher for malignant tumours (5.7 %) where the recurrent laryngeal nerve might be directly invaded [35]. A relatively lower 6.25 % proportion of recurrent laryngeal nerve injury was reported from a study conducted in Mali [36]. The reason for the lower rate of RLN injury should be further explored and adequately addressed. Airway obstruction (6.67 %) was one of the top five most common complications following thyroidectomy in Africa. This finding was higher than the Mexican study (0.79 %) [37]. However, this was lower than a research finding from Greece (13.5 %) [38]. The possible variation might be related to differences in sample size, patient demography, environment and clinical status of the patient. A study has also shown that airway obstruction following thyroidectomy can occur due to intraoperative bleeding, nerve injury, and laryngeal oedema [37].

According to the current systematic review and meta-analysis, wound site infections following thyroidectomy were reported to be 5.1 %. This finding was comparably higher than previous data from Brazil, indicating an incidence of 0–2 % [39]. This variation could be due to the use of inadequate sterility technique.

In this systematic review and meta-analysis, a significant number of patients developed hypocalcemia (pooled prevalence), which was found to be 5.6 %. Similar findings were reported from a study conducted in Italy [40].

## Limitation

The current systematic review and meta-analysis did not include factors associated with thyroid disease. Only articles published in English were selected. Operational definitions may vary among the publications. The heterogeneity of the data makes generalization of the findings difficult.

## Conclusion

Toxic goiters were the predominant indications for thyroidectomy in low and middle income African countries. Sub-total thyroidectomy was the most practiced surgery for goiter in the region. The postoperative complication remains considerably higher than previous studies. Hypoparathyroidism was found to be a concerning complication.

## Recommendations

Based on the systematic review and meta-analysis findings, the concerned bodies should collaborate to prepare management protocols to handle thyroid pathologies in low and middle income African countries. Stakeholders should collaborate to provide logistic and essential support to African facilities to build standard operating and patient follow-up facilities for thyroid disease. General surgeons should be trained in refreshing thyroid surgery to deal with it as endocrine surgeons. Iodine supplementation should be monitored continuously to assess for possible side effects. The reasons for higher post-thyroidectomy complications should be looked at, explored, and addressed.

## CRediT authorship contribution statement

**Bekalu Getachew:** Writing – review & editing, Writing – original draft, Visualization, Validation, Software, Methodology, Investigation, Formal analysis, Data curation, Conceptualization. **Mekbeb Afework:** Writing – review & editing, Validation, Supervision, Formal analysis, Conceptualization. **Girmaye Tamrat:** Writing – review & editing, Visualization, Supervision, Software, Methodology, Data curation.

## Consent for publication

Not applicable.

## Ethics approval and consent to participate

Not applicable.

## Funding

The author(s) received no financial support for the research, authorship, and/or publication of this article.

## Declaration of competing interest

The author(s) declared no potential conflicts of interest with respect to the research, authorship, and/or publication of this article.

## Data Availability

Data will be available upon request of the corresponding author.
